# Reference-free determination of tissue absorption coefficient by modulation transfer function characterization in spatial frequency domain

**DOI:** 10.1186/s12938-017-0394-z

**Published:** 2017-08-08

**Authors:** Weiting Chen, Huijuan Zhao, Tongxin Li, Panpan Yan, Kuanxin Zhao, Caixia Qi, Feng Gao

**Affiliations:** 1 College of Precision Instrument and Optoelectronics Engineering, Tianjin University, Tianjin, 300072 China; 2 Tianjin Key Laboratory of Biomedical Detecting Techniques and Instruments, Tianjin, 300072 China

**Keywords:** Determination of absorption coefficient, Spatial frequency domain measurement, Modulation transfer function, Reference-free measurement

## Abstract

**Background:**

Spatial frequency domain (SFD) measurement allows rapid and non-contact wide-field imaging of the tissue optical properties, thus has become a potential tool for assessing physiological parameters and therapeutic responses during photodynamic therapy of skin diseases. The conventional SFD measurement requires a reference measurement within the same experimental scenario as that for a test one to calibrate mismatch between the real measurements and the model predictions. Due to the individual physical and geometrical differences among different tissues, organs and patients, an ideal reference measurement might be unavailable in clinical trials. To address this problem, we present a reference-free SFD determination of absorption coefficient that is based on the modulation transfer function (MTF) characterization.

**Methods:**

Instead of the absolute amplitude that is used in the conventional SFD approaches, we herein employ the MTF to characterize the propagation of the modulated lights in tissues. With such a dimensionless relative quantity, the measurements can be naturally corresponded to the model predictions without calibrating the illumination intensity. By constructing a three-dimensional database that portrays the MTF as a function of the optical properties (both the absorption coefficient *μ*_*a*_ and the reduced scattering coefficient $\mu^{\prime}_{s}$) and the spatial frequency, a look-up table approach or a least-square curve-fitting method is readily applied to recover the absorption coefficient from a single frequency or multiple frequencies, respectively.

**Results:**

Simulation studies have verified the feasibility of the proposed reference-free method and evaluated its accuracy in the absorption recovery. Experimental validations have been performed on homogeneous tissue-mimicking phantoms with *μ*_*a*_ ranging from 0.01 to 0.07 mm^−1^ and $\mu^{\prime}_{s}$ = 1.0 or 2.0 mm^−1^. The results have shown maximum errors of 4.86 and 7% for $\mu^{\prime}_{s}$ = 1.0 mm^−1^ and $\mu^{\prime}_{s}$ = 2.0 mm^−1^, respectively. We have also presented quantitative ex vivo imaging of human lung cancer in a subcutaneous xenograft mouse model for further validation, and observed high absorption contrast in the tumor region.

**Conclusions:**

The proposed method can be applied to the rapid and accurate determination of the absorption coefficient, and better yet, in a reference-free way. We believe this reference-free strategy will facilitate the clinical translation of the SFD measurement to achieve enhanced intraoperative hemodynamic monitoring and personalized treatment planning in photodynamic therapy.

## Background

Recently, the spatial frequency domain (SFD) measurement has attracted increasing interests since it allows rapid and non-contact wide-field imaging of tissue optical properties [1–5]. Compared to the traditional near-infrared imaging modalities in epi-illumination mode, the emerging modality features scan-free wide-field illumination together with mesoscopic-scale detection, all benefiting from the implementation of the spatial modulated excitation. The advantages of the SFD mode lend itself well suited for imaging skin tissues during photodynamic therapy [6–10]. Specifically, by measuring the tissue absorption coefficients at two or more wavelengths, quantitative mapping of the concentrations of the tissue chromophores (dominant by oxy- and deoxy-hemoglobins in the near-infrared window ~600–900 nm) is achieved [11–13], and thus hemodynamic and oxygenation status as well as vascular distribution for disease diagnosing, staging and therapeutic response assessment are obtained.

In 1998, Dognitz and Wagnières reported the first use of the SFD method for measuring the tissue optical properties [14]. A wide-field light source modulated with radially-varying square wave was employed and both the diffuse reflectivity and the modulation depth of the backscattering light were used to recover the optical properties at a single point in space. In 2005, Cuccia et al. proposed a single-frequency sinusoidal modulation based imaging, where images of the direct current (DC) and alternating current (AC) components of the modulated reflectance were simultaneously extracted using a phase-shifting demodulation technique, and pixel-by-pixel recovery of the optical properties was achieved from joint use of the AC and DC amplitudes [2, 15]. This approach has been a mainstream technique for SFD imaging. Since then, further studies on improved techniques such as the fast demodulation and depth-resolved recovery, etc., as well as clinical applications, have been comprehensively reported [16–23].

Nevertheless, to our knowledge, all the reported SFD methods require a reference measurement to calibrate the mismatch between the absolute intensity and the model predictions of the diffuse reflectance. According to the explanations in Ref. 15 (Eqs. (22) and (23)), a reference phantom with the known optical properties must be measured within the same experimental setup and physical condition as those for the test one. In practice, this consistency can be individually affected by the net incident fluence, which is relevant to both the optical reflectivity and geometrical morphology of sample surface, as well as by the system modulation transfer function (MTF), which varies with the object-image distance at each spatial location [24–26]. Therefore, it is usually difficult to prepare a universal reference measurement in clinical trials.

To address the adversity, we attempt in this study a reference-free SFD method for the determination of the optical properties. Instead of using the absolute reflectance amplitude, we herein employ the MTF to characterize the propagation of the modulated lights in tissue. This dimensionless relative quantity naturally enables correspondence of the SFD measurements to the model predictions without the intensity calibration. Through the establishment of a database that links the MTF to the tissue optical properties (both the absorption coefficient *μ*_*a*_ and the reduced scattering coefficient $\mu^{\prime}_{s}$) and the modulation frequency (ranges from 0.05 to 0.30 mm^−1^), the optical properties can be theoretically retrieved using the SFD measurement at one or more frequencies.

It is widely believed that separating the scattering from the absorption in SFD requires at least one high-frequency modulation (typically at the spatial frequency of f > 0.5 mm^−1^), with the high-frequency response dominant by scattering and the low-frequency response dominant by both absorption and scattering [1, 4, 27]. However, due to the low-pass feature of high-scattering media such as tissues, intensity of the modulated diffuse reflectance attenuates severely as the modulation frequency increases, leading to great difficulties and complexities in detecting high-frequency modulated reflectance. In addition, the MTF, as a relative measure, also lacks the constraints on the absolute amplitude of the frequency response, and thus can further aggravate the inverse coupling between the absorption and scattering as the modulation frequency is not high enough. In view of the fact, we assume in the remainder of this manuscript a priori knowledge of the reduced scattering coefficient, and focus on the absorption-only determination. The practicability of the assumption have been justified in some of the clinical applications such as intraoperative monitoring of tissue oxygenation, where tracking the dynamic variations in oxygen metabolism is a primary concern and requires fast and convenient determination of absorption coefficient, while the scattering background is reasonably regarded as a constant and easy to pre-determinate using the space- or time-resolved spectroscopy [28, 29].

## Methods

### Modulation transfer function

We begin with assuming a linear optical medium illuminated by a spatially modulated light in the x-direction of the Cartesian coordinate system. The SFD expression of the modulated source fluence is given as $S(f) = A_{S}^{(0)} \delta (f) + A_{S}^{{(f_{x} )}} \delta (f - f_{x} )$, with $A_{S}^{\left( 0 \right)}$ and $A_{S}^{{(f_{x} )}}$ being the amplitude of the DC component and the amplitude of the AC component at the modulation frequency *f*_*x*_, respectively. The modulated source light gives rise to a reflected diffuse photon fluence modulated at the same frequency, with its SFD expression correspondingly given as $R(f) = A_{R}^{(0)} \delta (f) + A_{R}^{{(f_{x} )}} \delta (f - f_{x} )$, with $A_{R}^{\left( 0 \right)}$ and $A_{R}^{{(f_{x} )}}$ being the DC and AC amplitudes, respectively.

By definition, the medium MTF at the modulation frequency is experimentally calculated as the ratio of the modulation depth in reflected fluence to that in the source, i.e.,

1 $$MTF(f_{\text{x}} ) = \frac{{M_{R} (f_{x} )}}{{M_{S} (f_{x} )}}$$

where *M*_*S*_ and *M*_*R*_ denote the modulation depths of the source and reflected fluence, respectively, i.e., $M_{S} (f_{x} ) = A_{S}^{{(f_{x} )}} /A_{S}^{(0)}$ and $M_{R} (f_{x} ) = A_{R}^{{(f_{x} )}} /A_{R}^{(0)}$.

In the real spatial domain, the spatial response of medium to excitation of a high-contrast line beam, $R_{d}^{LSF} \left( x \right)$, is defined as the line spread function (LSF), and specified in the SFD by its one-dimension (1-D) Fourier transformation, referred to as the system transfer function (STF), i.e., $H\left( {f_{\text{x}} } \right) = \int_{ - \propto }^{ + \propto } {R_{d}^{LSF} \left( x \right) { \exp } \left( { - j 2\pi f_{\text{x}} x} \right)dx}$. The SFD-STF can be simply calculated as the ratio of the output AC amplitude to the input one, i.e., $H(f_{x} ) = A_{R}^{{(f_{x} )}} /A_{S}^{{(f_{x} )}}$. Accordingly, Eq. (1) can be also given in the form of the SFD-STF

2 $$MTF(f_{x} ) = \frac{{H(f_{x} )}}{{H(f{ = }0)}}$$

Note that although it is physically meaningless to define the MTF for the DC case, it converges to 1 as *f*_*x*_ approaches to 0 in terms of Eq. (2). This implies that the MTF calculation can be extended to include the scenario of zero modulation frequency when necessary. Equation (2) is the basis for the following three-dimension (3-D) database construction.

### Construction of 3-D MTF database

In terms of Eq. (2), the MTF is essentially calculated as the normalized Fourier series expansion of the real spatial domain LSF. Therefore, the conventional photon propagation models, such as the Monte Carlo (MC) simulation, radiative transfer equation and its approximations, can be applied to predicting the MTF through the LSF calculation. Based on the reported optical properties of human skin and subcutaneous tissue [30, 31], and meanwhile taking into account the sensitivity of the prototype SFD measuring system, we calculate in this study the MTF in the spatial frequency range of 0.05 to 0.30 mm^−1^, for *μ*_*a*_ ranging from 0.005 to 0.100 mm^−1^ at a step of 0.005 mm^−1^ and $\mu^{\prime}_{s}$ ranging from 0.4 to 2.2 mm^−1^ at a step of 0.2 mm^−1^, while keeping a constant anisotropy factor of *g* = 0.9. The MTF values for all combinations of the 20 absorption coefficients and the 10 reduced scattering coefficients are obtained by the following steps: Firstly, the spatially resolved diffuse reflectance of tissue for a single-point incidence $R_{d}^{PSF} \left( {x, y} \right)$, referred to as the point spread function (PSF), is predicted at a spatial resolution of 0.1 mm, using the steady-state MC simulation, with 10^8^ photons injected [32]. Secondly, the LSF $\left( {R_{d}^{LSF} \left( x \right)} \right)$ is deduced from convoluting the PSF with the distribution function of a line source that extends infinitely in y-direction, simply achieved by binning (summing up) the 2-D response along the y-direction. Thirdly, the MTF is calculated according to Eq. (2) in the frequency range of 0.05–0.3 mm^−1^ at a step of 0.01 mm^−1^, by Fourier transforming $R_{d}^{PSF} \left( x \right)$. Since our MC simulations have demonstrated that the intensity of the diffuse reflectance at a site 50 mm far from the source is below the shot noise level for all the above combinations of *μ*_*a*_ and $\mu^{\prime}_{s}$, the LSF calculation is truncated to a data length of 999 for the Fourier transform. In this way, a database of the MTF curves that correspond to the all 200 pairs of the optical properties can be established. Finally the linear interpolation procedure is applied to the MTF curves on a finer *μ*_*a*_-grid, to enhance the resolution of the *μ*_*a*_-determination to 0.001 mm^−1^.

**Fig. 1:**
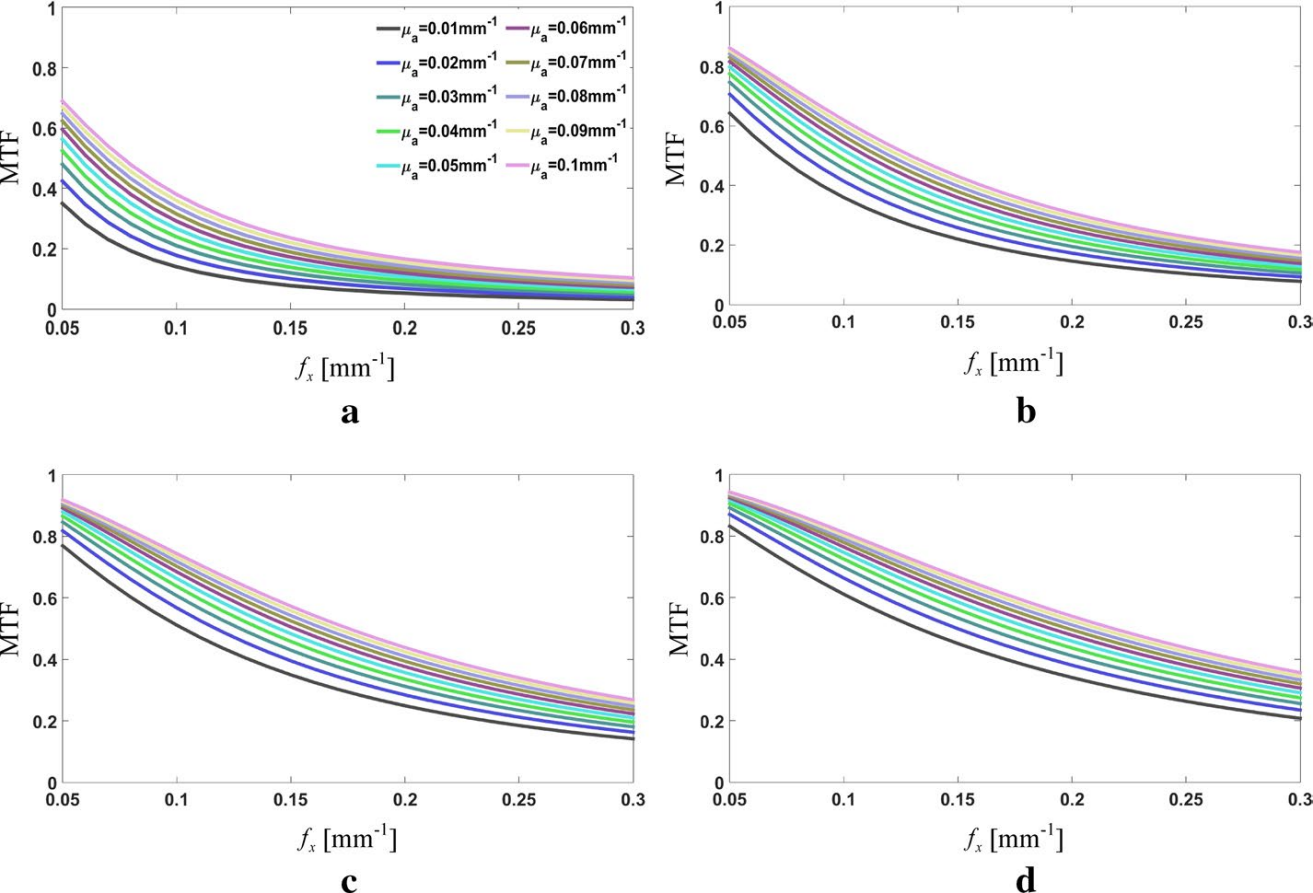
MTF calculations for a varying *μ*_*a*_ from 0.01 to 0.10 mm^−1^ at **a** $\mu^{\prime}_{s}$ = 0.4 mm^−1^, **b** $\mu^{\prime}_{s}$ = 1.0 mm^−1^, **c** $\mu^{\prime}_{s}$ = 1.6 mm^−1^, and **d** $\mu^{\prime}_{s}$ = 2.2 mm^−1^

**Fig. 2:**
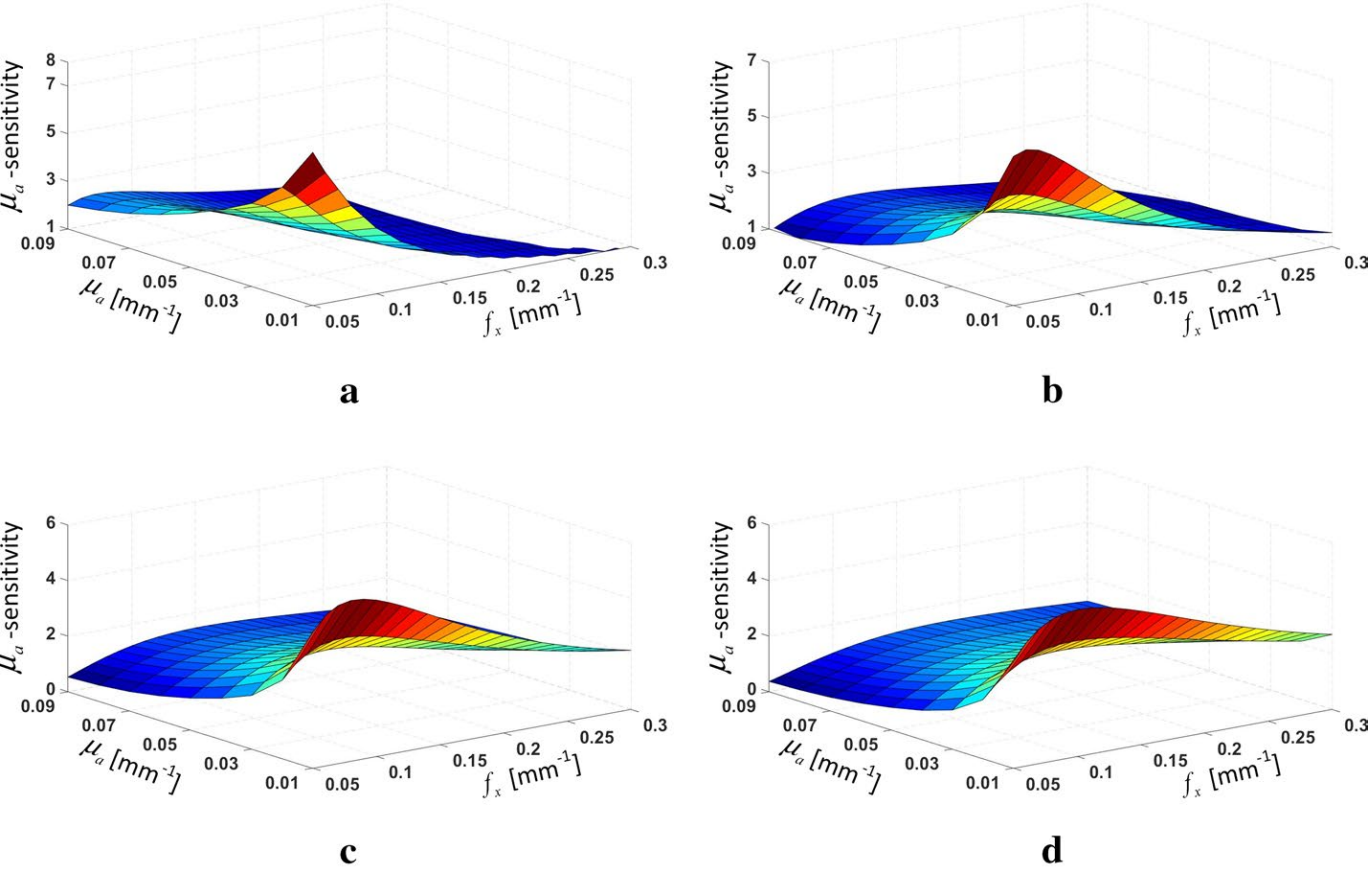
The *μ*_*a*_-sensitivity calculations at **a** $\mu^{\prime}_{s}$ = 0.4 mm^−1^, **b** $\mu^{\prime}_{s}$ = 1.0 mm^−1^, **c** $\mu^{\prime}_{s}$ = 1.6 mm^−1^, and **d** $\mu^{\prime}_{s}$ = 2.2 mm^−1^

As examples, the MTF curves for varying *μ*_*a*_ are illustrated in Fig. 1, at four fixed $\mu^{\prime}_{s}$ values of 0.4, 1.0, 1.6 and 2.0 mm^−1^, respectively. The calculations manifest the low-pass filtering effect of tissue on spatially modulated lights, where all the MTFs drop off with the increase in the spatial frequency but at a slowing-down slop as the scattering increases, and intuitively demonstrates the feasibility of using the MTF for the *μ*_*a*_-determination. In addition, it is shown in Fig. 1 that the MTF curves shift up on whole with the increase in *μ*_*a*_, indicating an increase in the modulation depth of the diffuse reflectance with the increase in *μ*_*a*_, and thus inferring an more significant effect of *μ*_*a*_ on the DC attenuation than the AC one. A further investigation, as shown in Fig. 2, analogously calculates the sensitivity of the MTF to the absorption coefficient (the *μ*_*a*_-sensitivity), simply defined as the ratio of the MTF change to the absorption change, at the aforementioned set of $\mu^{\prime}_{s}$. It is shown that the *μ*_*a*_-sensitivity trends to achieve a higher value at lower absorption, and decreases on whole with the maximum moving toward the direction of frequency increase as $\mu^{\prime}_{s}$ increases. This observations are implicitly in accordance with the past findings that the low frequency component is more sensitive to variations in *μ*_*a*_ while the high frequency component is more sensitive to variations in $\mu^{\prime}_{s}$, and could presage an optimized selection of the working frequency according to the $\mu^{\prime}_{s}$-range [1, 18, 27].

### Inversion methods

As a priori $\mu^{\prime}_{s}$ is available, only one MTF measurement at a single frequency is sufficient to determine *μ*_*a*_ from the established database simply by a look-up table method. To enhance the noise robustness, the inversion scheme can be generalized to the following least square optimization for multi-frequency measurements.

3 $$\mathop {\hbox{min} }\limits_{{\mu_{a} }} \sum\limits_{n = 1}^{N} {\{ MTF_{m} [f_{x}^{\left( n \right)} ] - MTF[\mu_{a} ,f_{x}^{\left( n \right)} ]\}^{2} }$$

where $MTF_{m} \left[ {f_{x}^{(n)} } \right]$ and $MTF\left[ {\mu_{a} , f_{x}^{(n)} } \right]$ are the measured and model-predicted MTFs, respectively, with the latter rapidly calculated by interpolating the database; $f_{x}^{(n)}$ $\left( {n = 1,{ 2}, \ldots ,N} \right)$ is the *n*-th spatial frequency. Owing to the monotonic dependence of the MTF on the absorption coefficient, the above minimization can be uniquely achieved when *μ*_*a*_ approaching to the true values. Obviously, by employing the multi-frequency optimization of the MTFs, the noise influence in the single measurement on the *μ*_*a*_-determination can be greatly alleviated thanks to the averaging effect of the least-square fitting. While the multi-frequency optimization provides more robust determination of *μ*_*a*_ than the look-up table method does, it is essential in practice to make a compromise between the determination accuracy and the measurement cost.

For homogeneous optical media, the diffuse reflectance will ideally maintain the modulation frequency without spectrum broadening. In this case, a composited illumination of multi-frequency modulation patterns is employed and the MTFs at multiple frequencies are extracted from the single snapshot reflectance image using the Fourier frequency spectrum analysis, and determine the bulk *μ*_*a*_ using the least-square curve-fitting method. To reduce the truncation errors, the raw data is weighted by a Blackman window function prior to the Fourier transform. The Blackman window is widely believed to be excellent in estimating the amplitude-frequency characteristics but suffers from degraded spectral resolution. As a result, a minimal frequency spacing should be assured for reliably extracting the multiple frequency components from the raw data, according to the field of view (FOV) and the spatial resolution of the system.

For inhomogeneous optical medium, due to adverse effect of the frequency spectrum broadening of the diffuse reflectance on selection of the multiple modulation frequencies, a successive illumination of multi-frequency patterns is used to avoid the spectrum aliasing. To map the *μ*_*a*_-distribution, the raw data successively measured at each frequency is then demodulated in a pixel-by-pixel fashion by employing the three-phase amplitude demodulation technique [2]

4 $$A^{{(f_{x}) }} (x_{i} ) = \frac{{2^{1/2} }}{3}\{ [I_{1} (x_{i} ) - I_{2} (x_{i} )]^{2} + [I_{2} (x_{i} ) - I_{3} (x_{i} )]^{2} + [I_{3} (x_{i} ) - I_{1} (x_{i} )]^{2} \}^{1/2}$$

5 $$A^{(0)} (x_{i} ) = \frac{1}{3}[I_{1} (x_{i} ) + I_{2} (x_{i} ) + I_{3} (x_{i} )]$$

where *x*_*i*_ is the position of the *i*-th pixel; *I*_1_, *I*_2_ and *I*_3_ are the measured reflectance images at modulation frequency of *f*_*x*_ with the phase offsets of 0, 2*π*/3 and 4*π*/3, respectively.

### Prototype SFD system

**Fig. 3:**
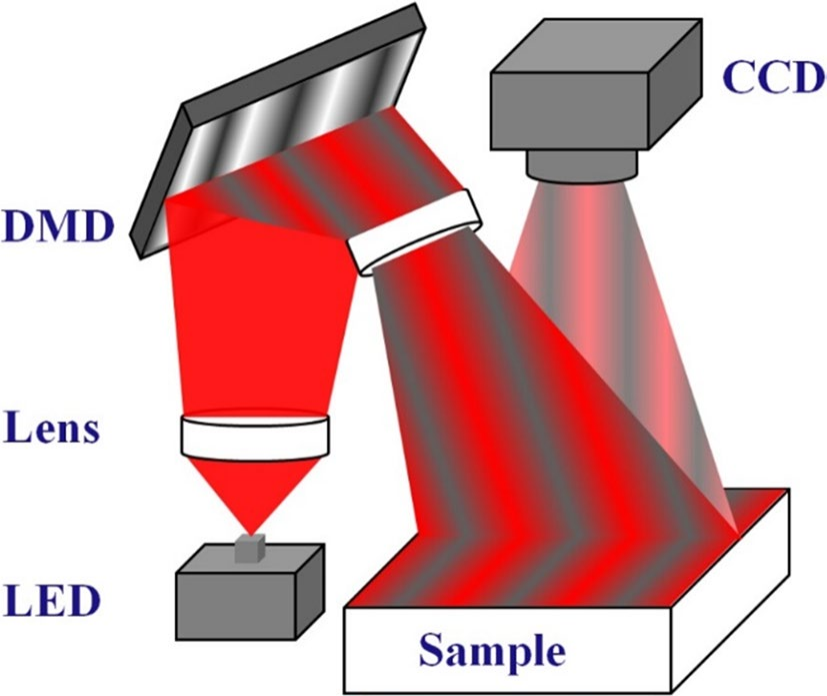
Schematic of the prototype SFD measuring system

The setup of the used prototype SFD measuring system is shown in Fig. 3. Light emitted from a light-emitting diode (LED) source at the wavelength of 660 nm (M660F1, Thorlabs, USA) is expanded and then coupled to a digital micromirror device (DMD) (LightCrafter 4500, Texas Instruments, USA) by a lens system. The spatial modulated patterns on the DMD are formed by assigning an 8-bit value (0–255) to each micromirror with customized control software. To reduce the specular reflection artifact, the spatially modulated light is projected onto a sample with a slight angle (≈3°) relative to the normal of the sample. Diffuse reflectance images of 51.2 mm × 51.2 mm are captured by a 16-bit, 512 × 512 pixel charge coupled device (CCD) camera (Rolera-MGi Plus, QImaging, Canada) placed right above the sample. This configuration leads to an approximate sampling rate of 0.1 mm and a raw data length of 512, and accordingly, a minimum frequency spacing of 0.06 mm^−1^ should be assured as the composited illumination of multi- frequency patterns is to be applied.

### System calibration

In a realistic scenario, the overall MTF of the measurement is the combined contribution from both the MTF of the sample, *MTF*_*sample*_(*f*_*x*_), and that of the measuring system (both the DMD and CCD), *MTF*_*system*_(*f*_*x*_). Thus, the modulation depth of the measured reflectance, *M*_*R*_(*f*_*x*_), is given by

6 $$M_{R} (f_{x} ) = M_{I} (f_{x} ) \cdot MTF_{sample} (f_{x} ) \cdot MTF_{system} (f_{x} )$$

where *M*_*I*_(*f*_*x*_) is the known modulation depths of the input to the DMD. To obtain *MTF*_*system*_(*f*_*x*_), a BaSO_4_ plate which has been widely used as a 100% reflectance standard is illuminated with the modulated source, and the modulation depth of its reflectance, *M*_*P*_(*f*_*x*_), is measured. We then get

7 $$MTF_{system} ( {f_{x} } ) = M_{P} ( {f_{x} })/M_{I} ( {f_{x} })$$

Now, with *M*_*I*_(*f*_*x*_) and *MTF*_*system*_(*f*_*x*_) available, the genuine sample MTF required for the *μ*_*a*_-determination, *MTF*_*sample*_(*f*_*x*_), can be readily calculated from Eq. (6).

## Results and discussion

To validate the proposed method, simulation, phantom and experimental investigations were performed.

### Simulation validations

The simulation validation was conducted on a total of 36 homogeneous samples, with their optical properties coming from 36 combinations of *μ*_*a*_ = 0.01, 0.02, …, 0.09 mm^−1^ and $\mu^{\prime}_{s}$ = 0.4, 1.0, 1.4, 2.0 mm^−1^, are tested. A composited illumination of multi-frequency modulation patterns at frequencies of 0.07, 0.13, 0.19, and 0.25 mm^−1^ was used. The spatial domain diffuse reflectance for each sample was simulated with the following steps: Firstly, the LSF of each sample was generated following steps 1 and 2 in “Construction of 3-D MTF database”, except that the number of the photons being run in the MC simulation was 10^6^ for the purpose of increasing the shot noise level. Secondly, the LSF was Fourier-transformed to extract the SFD responses at the DC and the four modulation frequencies, from which the real spatial domain response to the four-frequency modulated source was recovered using the inverse Fourier transform. Thirdly, the recovered data was corrupted by a 20 dB Gaussian noise to emulate the realistic measurement.

**Fig. 4:**
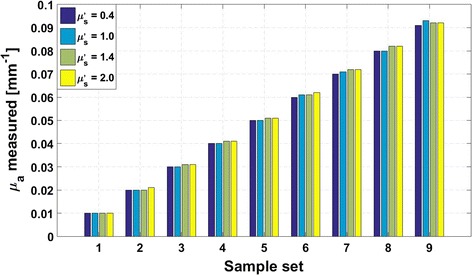
Simulative determination of the absorption coefficient from a composited illumination of multi-frequency modulation patterns at frequencies of 0.07, 0.13, 0.19, and 0.25 mm^−1^. Sample set 1–9 specify the samples with *μ*_*a*_ = 0.01, 0.02, 0.03, 0.04, 0.05, 0.06, 0.07, 0.08, and 0.09 mm^−1^, respectively

For the inversion, the Fourier frequency spectrum analysis was firstly employed, and then *μ*_*a*_ of each sample was extracted from the four measured MTFs using the least-square curve-fitting method, with a priori knowledge on $\mu^{\prime}_{s}$. The results are shown in Fig. 4, where samples are divided into 9 groups according to the true values of *μ*_*a*_. It is observed that, with increase of *μ*_*a*_, the discrepancy between the true and measured *μ*_*a*_-values gradually increases, as a result of decreased *μ*_*a*_-sensitivity, with a maximum of 0.003 mm^−1^ occurred at a sample case of *μ*_*a*_ = 0.09 mm^−1^ and $\mu^{\prime}_{s}$ = 1.0 mm^−1^.

**Fig. 5:**
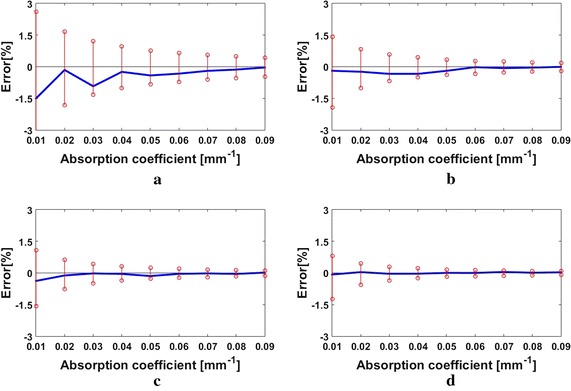
Comparisons between the MTF-ETRs (*red stem*) for the *μ*_*a*_-determination and the measurement errors of the simulated MTFs (*blue line*) for the reduced scattering coefficients of **a** $\mu^{\prime}_{s}$ = 0.4 mm^−1^, **b** $\mu^{\prime}_{s}$ = 1.0 mm^−1^, **c** $\mu^{\prime}_{s}$ = 1.4 mm^−1^, and **d** $\mu^{\prime}_{s}$ = 2.0 mm^−1^ at the spatial frequency of *f*_*x*_ = 0.07 mm^−1^

**Fig. 6:**
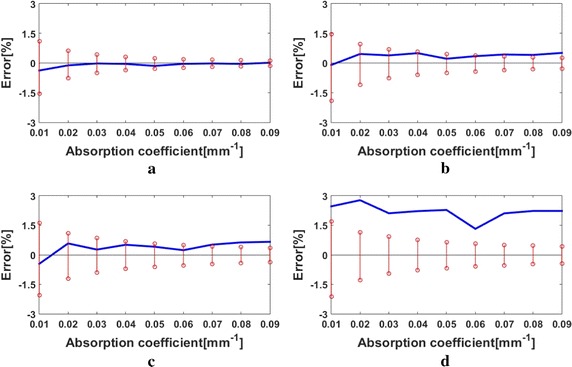
Comparisons between the MTF-ETRs (*red stem*) and the measurement errors of the simulated MTFs (*blue line*) for the reduced scattering coefficient of $\mu^{\prime}_{s}$ = 1.4 mm^−1^ at the spatial frequencies of **a** *f*_*x*_ = 0.07 mm^−1^, **b** *f*_*x*_ = 0.13 mm^−1^, **c** *f*_*x*_ = 0.19 mm^−1^, and **d** *f*_*x*_ = 0.25 mm^−1^

The accuracy of the proposed MTF-characterization-based method for the *μ*_*a*_-determination is contingent on the measurement errors of MTFs as well as the error-tolerance of the inversion algorithm. We define the measurement error of the MTF as

8 $$e(\mu_{a} ,\mu^{\prime}_{s} ,f_{x} ) = [MTF_{M} (\mu_{a} ,\mu^{\prime}_{s} ,f_{x} ) - MTF_{T} (\mu_{a} ,\mu^{\prime}_{s} ,f_{x} )]/MTF_{T} (\mu_{a} ,\mu^{\prime}_{s} ,f_{x} )$$

where $MTF_{M} (\mu_{a} ,\mu^{\prime}_{s} ,f)$ and $MTF_{T} (\mu_{a} ,\mu^{\prime}_{s} ,f)$ are the measured and the true sample MTFs at the spatial frequency *f*_*x*_, respectively. To assess the influence of the measurement errors of the sample MTF on the look-up table scheme of the *μ*_*a*_-determination, we define the up and down error tolerance

9 $$d_{U} (\mu_{a}^{k} ,\mu^{\prime}_{s} ,f_{x} ) = [MTF(\mu_{a}^{k + 1} ,\mu^{\prime}_{s} ,f_{x} ) - MTF(\mu_{a}^{k} ,\mu^{\prime}_{s} ,f_{x} )]/MTF(\mu_{a}^{k} ,\mu^{\prime}_{s} ,f_{x} )$$

10 $$d_{D} (\mu_{a}^{k} ,\mu^{\prime}_{s} ,f_{x} ) = [MTF(\mu_{a}^{k - 1} ,\mu^{\prime}_{s} ,f_{x} ) - MTF(\mu_{a}^{k} ,\mu^{\prime}_{s} ,f_{x} )]/MTF(\mu_{a}^{k} ,\mu^{\prime}_{s} ,f_{x} )$$

where $\mu_{a}^{k}$ is the *k*-th absorption coefficient in the *μ*_*a*_-grid with $\mu_{a}^{k} < \mu_{a}^{k + 1}$. It is inferred from Fig. 1 that *d*_*U*_ ≥ 0 and *d*_*D*_ ≤ 0. Assuming that the MTFs for a fixed $\mu^{\prime}_{s}$ vary as a linear function of *μ*_*a*_, the look-up table method can accurately determine *μ*_*a*_ provided that the condition $d_{D} (\mu_{a} ,\mu^{\prime}_{s} ,f_{x} )/2 \le e(\mu_{a} ,\mu^{\prime}_{s} ,f_{x} ) \le d_{U} (\mu_{a} ,\mu^{\prime}_{s} ,f_{x} )/2$ is satisfied. In this view, we defined the interval $[d_{D} (\mu_{a} ,\mu^{\prime}_{s} ,f_{x} )/2,\,d_{U} (\mu_{a} ,\mu^{\prime}_{s} ,f_{x} )/2]$ as the error-tolerance range (ETR) of a measured MTF for the *μ*_*a*_-determination. Figure 5 contrasts the MTF-ETRs with the measurement errors of the simulated MTFs for $\mu^{\prime}_{s}$ = 0.4, 1.0, 1.4, 2.0 mm^−1^ at the spatial frequency of *f*_*x*_ = 0.07 mm^−1^, and Fig. 6 compares the MTF-ETRs and the measurement errors of the simulated MTFs for $\mu^{\prime}_{s}$  = 1.4 mm^−1^ at the spatial frequencies of *f*_*x*_ = 0.07, 0.13, 0.19, 0.25 mm^−1^.

In Fig. 5, the MTF-ETRs decrease with the increase in $\mu^{\prime}_{s}$, due to the decrease in the *μ*_*a*_-sensitivity and the increase in the MTF value, and the measurement errors of the MTFs decrease conformably, indicating no clear correlation between $\mu^{\prime}_{s}$ and the accuracy of the *μ*_*a*_-determination. It is noted that in our simulations, the SNR is set to be the same for all the measured reflectance without regard to $\mu^{\prime}_{s}$. But in practice, the SNR of the measured diffuse reflectance might increase with the increase in $\mu^{\prime}_{s}$, owing to the increased intensity of the diffuse reflectance. Therefore it is expected that accuracy of the *μ*_*a*_-determination could be improved with increasing $\mu^{\prime}_{s}$.

In Fig. 6, with the increase in the spatial frequency, it is observed that both the ETRs and the measurement errors of MTFs trend to increase. As the spatial frequency reaches 0.25 mm^−1^, the measurement errors of the MTFs are all beyond the corresponding ETRs. This implies that a look-up-table-method-based retrieval of *μ*_*a*_ at this frequency would be inaccurate. A reason for the degraded performance with the increase in the spatial frequency is that the high frequency components account for little of the whole spatial response, and thus suffer from deteriorated SNRs. It is thus concluded that low frequency modulation is more preferable as the look-up table method is employed.

### Phantom experiments

Experiments were conducted on liquid tissue-simulating phantoms consisting of deionized water as the diluent, India ink as the absorber and Intralipid-10% as the scatterer. According to the spectral absorbance of a pre-prepared diluted India ink measured by a spectrophotometer (UV2550, Shimadzu, Japan), and the reported scattering coefficient and the anisotropy of Intralipid-10% [33], a set of the optical properties were obtained from 14 combinations of *μ*_*a*_ = 0.01, 0.02, 0.03, 0.04, 0.05, 0.06, 0.07 mm^−1^ and $\mu^{\prime}_{s}$ = 1.0, 2.0 mm^−1^ for the phantoms. A semi-infinite plane geometry was realized by a tank with a length of 120 mm, a width of 120 mm, and a height of 50 mm. The tank was painted black to reduce the wall reflection.

Each sample was illuminated by composited three-frequency modulation patterns with three frequency sets of *f*_*x*,1_ = {0.06, 0.14, 0.22 mm^−1^}, *f*_*x*,2_ = {0.08, 0.16, 0.24 mm^−1^} and *f*_*x*,3_ = {0.10, 0.18, 0.26 mm^−1^}. The diffuse reflectance images corresponding to the three frequency sets were captured successively, and then summed up along the y-direction for 1-D Fourier transform along the x direction. Finally, the MTFs of each sample at the 9 modulation frequencies were calculated by Eq. (9).

**Fig. 7:**
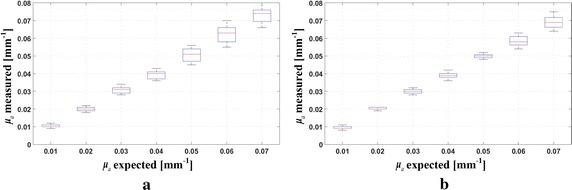
Boxplot graphs of the retrieved absorption coefficients for **a** $\mu^{\prime}_{s}$ = 1.0 mm^−1^ and **b** $\mu^{\prime}_{s}$ = 2.0 mm^−1^

For the *μ*_*a*_-determination, we recombined the 9 measured MTFs of each sample into three sets, i.e., the low-frequency set: LF = {*MTF*(*f*)|*f* = 0.06, 0.08, 0.10}, the middle-frequency set: *MF* = {*MTF*(*f*)|*f* = 0.14, 0.16, 0.18}, and the high-frequency set: *HF* = {*MTF*(*f*)|*f* = 0.22, 0.24, 0.26}, respectively. We thereafter retrieved 27 absorption coefficients using the least-square curve-fitting scheme, from 27 combinations of the three-frequency MTFs, with the MTFs in each combination coming from the LF, MF and HF, respectively. Boxplot graphs of the retrieved absorption coefficients are shown in Fig. 7a, b, for $\mu^{\prime}_{s}$ = 1.0 mm^−1^ and $\mu^{\prime}_{s}$ = 2.0 mm^−1^, respectively, with the relevant statistical and error analyses listed in Table 1.

**Table 1:** Statistical and error analyses of the retrieved absorption coefficients

Expected absorption coefficient ($\mu_{a}^{expect}$) [mm^−1^]	Mean ($\mu_{a}^{mean}$) [mm^−1^]		Variance [mm^−2^]		Error (*e*) [%]	
	$\mu^{\prime}_{s}$ = 1.0	$\mu^{\prime}_{s}$ = 2.0	$\mu^{\prime}_{s}$ = 1.0	$\mu^{\prime}_{s}$ = 2.0	$\mu^{\prime}_{s}$ = 1.0	$\mu^{\prime}_{s}$ = 2.0
0.0100	0.0104	0.0093	7.2 × 10^−7^	6.8 × 10^−7^	4.00	7.00
0.0200	0.0200	0.0203	1.9 × 10^−6^	5.1 × 10^−7^	0.00	1.50
0.0300	0.0310	0.0297	3.7 × 10^−6^	1.7 × 10^−6^	3.33	1.00
0.0400	0.0397	0.0388	6.0 × 10^−6^	2.8 × 10^−6^	0.75	3.00
0.0500	0.0507	0.0498	1.5 × 10^−5^	1.3 × 10^−6^	1.04	0.40
0.0600	0.0626	0.0584	2.3 × 10^−5^	7.2 × 10^−6^	4.33	2.67
0.0700	0.0734	0.0691	2.2 × 10^−5^	1.0 × 10^−5^	4.86	1.29

For the accuracy assessment of the *μ*_*a*_-determination, the relative error between the expected absorption coefficient, $\mu_{a}^{{expect}}$, and the mean of the measured absorption coefficient, $\mu_{a}^{mean}$, is used in the above table, i.e., $e = \left| {\left( {\mu_{a}^{mean} - \mu_{a}^{{expect}} } \right)/\mu_{a}^{{expect}}} \right|$.

Results in Table 1 demonstrate a maximum *μ*_*a*_-determination error of 4.86% for $\mu^{\prime}_{s}$ = 1.0 mm^−1^ and 7.00% for $\mu^{\prime}_{s}$ = 2.0 mm^−1^. A gradually increasing trend of the variance is observed as *μ*_*a*_ increases. This is probably due to the fact that as *μ*_*a*_ increase, the *μ*_*a*_-sensitivity decreased while the measurement errors of the MTFs increased. In contrast, as $\mu^{\prime}_{s}$ increases, decreased variance is observed in Table 1, which might be a result of the decreased measurement errors of the MTFs.

### Ex-vivo imaging of a subcutaneous tumor

**Fig. 8:**
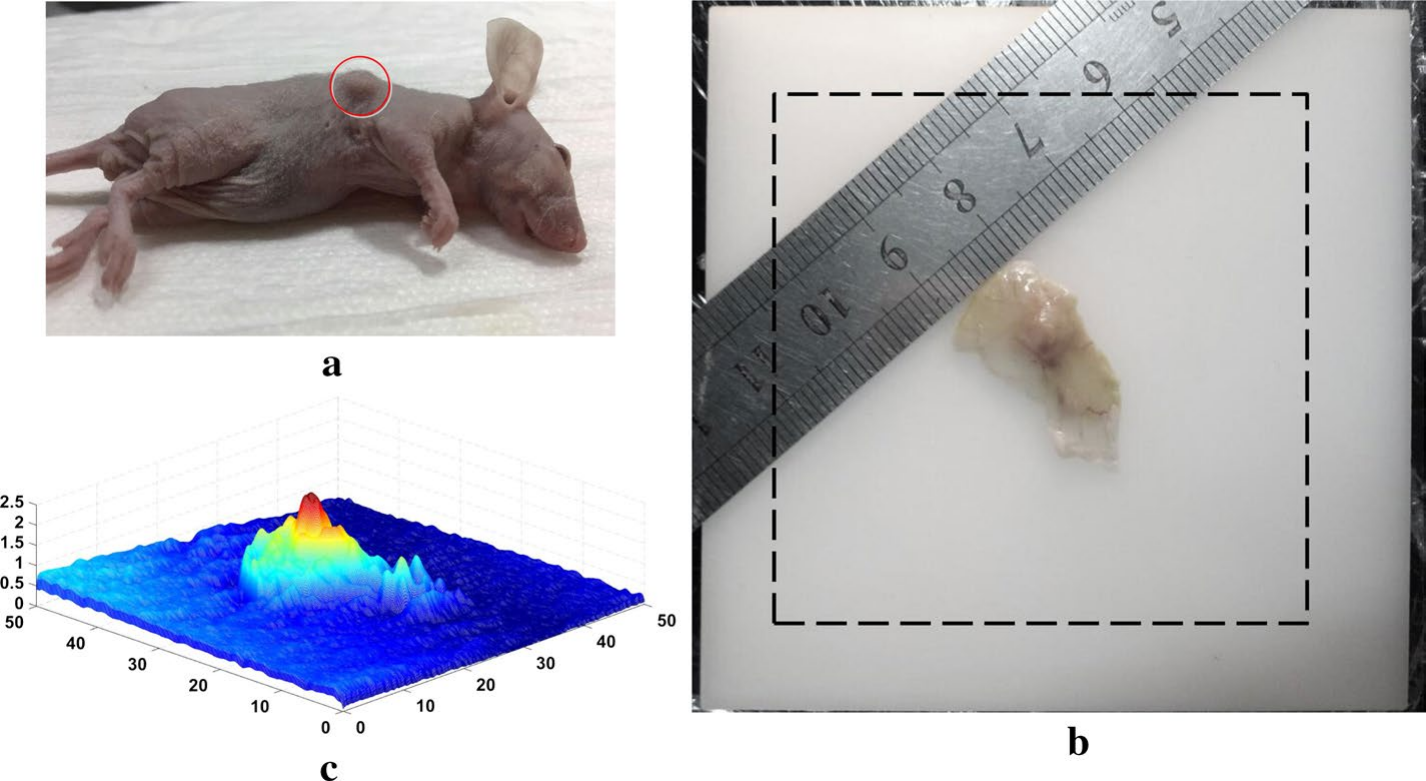
**a** A photograph of the subcutaneous xenografts mouse model, and the *red circle* indicates the location of the tumor; **b** the experimental sample consisting of the specimen and the phantom. The region marked with *black dotted square box* indicates the capture field (51.2 mm × 51.2 mm). **c** 3-D surface profile of the specimen

A subcutaneous xenograft model of human lung cancer (ATCC number: CCL-185) in a 4-week-old female nude mouse was investigated, as shown in Fig. 8. To obtain a reasonably flat sample, a specimen consisting of tumor, vessels, skin and subcutaneous tissues was cut out from the mouse and put on the upper surface of a solid tissue-mimicking phantom made from polyformaldehyde. The phantom was 80 mm in length, 80 mm in width and 40 mm in height, with *μ*_*a*_ = 0.0038 mm^−1^ and $\mu^{\prime}_{s}$ = 1.0 mm^−1^ according to the product manual. The size of the tumor was ~7 mm in largest dimension. The 3-D surface profile of the specimen was measured using the prototype system with the upper surface of the phantom being taken as the reference. The surface height map is reconstructed using a phase-shifting profilometry, and shown in Fig. 8c, with the maximum height of the tumor being approximated 2.4 mm [34].

**Fig. 9:**
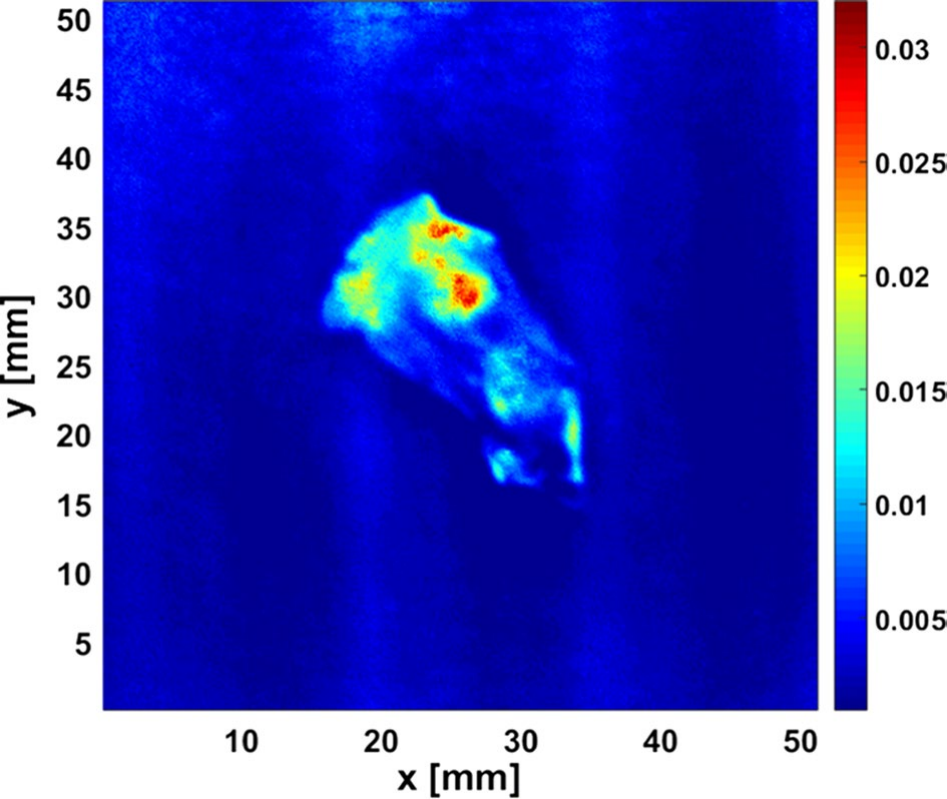
Reconstructed image of the specimen

To achieve spatially resolved imaging of the specimen, the sample was sequentially illuminated three times at the same modulation frequency of *f*_*x*_ = 0.06 mm^−1^ with the different phase offsets of 0, 2*π*/3, 4*π*/3, and the diffuse reflectance images were captured with the CCD camera in full 512 × 512 resolution. Profile corrections of the reflectance intensity using a multi-height calibration approach along with a Lambertian model were firstly conducted [35]. Then the corrected images were put into Eqs. (4) and (5) to extract both the DC and the AC modulated reflectance images, from which the MTF was calculated pixel-by-pixel. Finally, pixel-by-pixel *μ*_*a*_-determination is achieved using the look-up table method, with the assumption of $\mu^{\prime}_{s}$ = 1.0 mm^−1^ over the whole FOV. The reconstructed image is shown in Fig. 9.

In Fig. 9, we recognize an extremely high absorption region which is basically coincident with the tumor bulge observed in the surface height map, demonstrating the high absorption contrast of the tumor to the surrounding tissue as a result of tumor angiogenesis [36]. The circumambient high absorption region near the tumor suggests potential tumor invasion that is invisible to naked eyes, implying that the method may serve as a powerful tool for identifying tumor margins. It should be noted that up to now SFD imaging still suffers from degraded quantitation and spatial resolution, known as the partial volume effect, because the data acquired by each detector (i.e., each pixel on the CCD array) is analyzed independently of all the other detectors. As a result, in Fig. 9, the absorption coefficient of the specimen might be underestimated due to the averaging of the background, and image of the tumor target is slightly blurred due to the averaging of the neighboring volumes. Cuccia et al. have illustrated well about the lateral and depth-dependent partial volume effects in SFD imaging [15]. To solve the problem, we will work on the development of an accurate SFD forward model for inhomogeneous optical medium as well as a reconstruction method that employs rigorous sensitivity function of detector in the future.

## Conclusions

In summary, we have developed a novel approach based on the MTF characterization to achieve the reference-free determination of absorption coefficients. A 3-D database that depicts the MTF as a function of the absorption coefficient, the reduced scattering coefficient and the spatial frequency have been established, enables the inversion methods of both the least-square curve-fitting and the look-up table being adopted for *μ*_*a*_-determination. Simulation results have verified the feasibility of the method as well as evaluated its performance in *μ*_*a*_-determination. Experimental performance have been evaluated for *μ*_*a*_ ranging from 0.01 to 0.07 mm^−1^, and the maximum errors in *μ*_*a*_-determination are 4.86% for $\mu^{\prime}_{s}$ = 1.0 mm^−1^ and 7.00% for $\mu^{\prime}_{s}$ = 2.0 mm^−1^. The results suggest that the proposed method can be applied to the accurate determination of the bulk absorption coefficient of tissue, and better yet, in a reference-free way. We have also presented quantitative ex vivo imaging of human lung cancer in a subcutaneous xenograft mouse model, and the result indicates that this reference-free method will facilitate the clinical translation of SFD measurement toward the diagnosis, staging and prognosis of those diseases that are accompanied by significant changes in optical absorption, such as non-melanoma skin cancer and port-wine stain.

## Abbreviations

SFD: spatial frequency domain
MTF: modulation transfer function
DC: direct current
AC: alternating current
LSF: line spread function
1-D: one-dimension
STF: system transfer function
MC: Monte Carlo
PSF: point spread function
FOV: field of view
LED: light-emitting diode
DMD: digital micromirror device
CCD: charge coupled device
ETR: error-tolerance range
